# An effective response to respiratory inhibition by a *Pseudomonas aeruginosa* excreted quinoline promotes *Staphylococcus aureus* fitness and survival in co-culture

**DOI:** 10.1128/jb.00101-25

**Published:** 2025-10-20

**Authors:** Franklin Roman-Rodriguez, Nupur Tyagi, Jisun Kim, Dane Parker, Jeffrey M. Boyd

**Affiliations:** 1 Department of Biochemistry and Microbiology, Rutgers, The State University of New Jersey 242612, New Brunswick, New Jersey, USA; 2 Department of Pathology, Immunology and Laboratory Medicine, Center for Immunity and Inflammation, Rutgers New Jersey Medical School 12286, Newark, New Jersey, USA; University of Massachusetts Chan Medical School, Worcester, Massachusetts, USA

**Keywords:** SrrAB, Rex, respiration, HQNO, *Pseudomonas aeruginosa*, *Staphylococcus aureus*

## Abstract

*Pseudomonas aeruginosa* and *Staphylococcus aureus* are primary bacterial pathogens frequently isolated from the airways of cystic fibrosis patients. *P. aeruginosa* produces secondary metabolites that negatively impact the fitness of *S. aureus,* allowing *P. aeruginosa* to become the most prominent bacterium when the species are co-cultured. Some of these metabolites inhibit *S. aureus* respiration. SrrAB is a staphylococcal two-component regulatory system (TCRS) that responds to alterations in respiratory status to help *S. aureus* transition between fermentative and respiratory metabolisms. Using *P. aeruginosa* mutant strains and chemical genetics, we established that *P. aeruginosa* secondary metabolites, 2-heptyl-4-quinolone N-oxide (HQNO) in particular, inhibit *S. aureus* respiration, resulting in decreased SrrAB transcriptional output. Metabolomic analyses demonstrated that the ratio of NAD^+^ to NADH increased upon prolonged culture with HQNO. Consistent with this, the activity of the Rex transcriptional regulator, which senses and responds to alterations in the NAD^+^/NADH ratio, repressed *srrAB* promoter activity upon HQNO treatment. The presence of SrrAB increased fitness when cultured with HQNO and enhanced survival when challenged with *P. aeruginosa. S. aureus* strains with reduced ability to maintain redox homeostasis via fermentation had decreased fitness when challenged with HQNO and lower survival when challenged with *P. aeruginosa*. These findings led to a model wherein *P. aeruginosa* secreted HQNO inhibits *S. aureus* respiration, resulting in manipulation of the redox status in both the membrane and cytoplasm, altering the transcriptional activities of SrrAB and Rex, which promote fitness and survival by increasing carbon flux through fermentative pathways to maintain redox homeostasis.

**IMPORTANCE:**

Cystic fibrosis is a hereditary respiratory disease that predisposes patients to bacterial infections, often caused by *Staphylococcus aureus* and *Pseudomonas aeruginosa*. Secondary metabolites excreted by *P. aeruginosa* decrease *S. aureus* fitness during co-infection, ultimately eliminating it. The regulatory systems and mechanisms that *S. aureus* uses to detect and respond to these metabolites are unknown. The data presented demonstrate that two regulatory systems that are stimulated by alterations in membrane or cytosolic redox status respond to the *P. aeruginosa*-produced respiratory toxin 2-heptyl-4-quinolone N-oxide (HQNO) by increasing transcription of genes utilized for fermentation, thereby promoting fitness. This study describes interactions between these two bacterial pathogens that could be exploited to decrease pathogen burden in individuals living with cystic fibrosis.

Cystic fibrosis patients suffer from increased lung and airway mucus because of a dysfunctional cystic fibrosis transmembrane conductance regulator (CFTR) (1). CFTR helps transport chloride through cell membranes. When CFTR function is disrupted, fewer chloride ions are exported from epithelial cells, resulting in reduced water retention in mucus, making the mucus thicker and harder to clear, which in turn allows bacteria to accumulate (1). Cystic fibrosis patients often have polymicrobial lung infections, with *Staphylococcus aureus* and *Pseudomonas aeruginosa* being two of the most abundant bacteria (2). Methicillin-resistant *S. aureus* is found in around 20% of individuals with cystic fibrosis between the ages of 10–20 (2). *P. aeruginosa* is found in around 17% of patients, and 3.5% of these carry multi-antibiotic-resistant strains (2). *S. aureus* is most prevalent in children and teenagers, but as patients age, *P. aeruginosa* becomes the most prevalent, coinciding with worsening symptoms and lower lung activity (2–8). *P. aeruginosa* secretes various compounds that cause *S. aureus* cell lysis, inhibition of respiration, and iron starvation, allowing it to outcompete *S. aureus* (9–15). Co-culture of *P. aeruginosa* with *S. aureus* can select for *S. aureus* small colony variants (SCVs) (16). SCVs are respiration-deficient and rely on fermentation for energy conservation. A fermentative lifestyle increases lactate release, which in turn is used as a primary carbon source by *P. aeruginosa* during co-infection (10, 16, 17).

The secretion of multiple *P. aeruginosa* secondary metabolites is regulated by quorum-sensing systems. There are a total of four described quorum sensing (QS) systems in *P. aeruginosa*, with the *Pseudomonas* quinolone system (PQS) being central to the production of various virulence factors (18). The *pqsABCDE* operon is one of the three main gene clusters of the PQS system, with PqsR serving as the receptor and transcriptional regulator (18, 19). The PQS QS system is responsible for regulating the production of several virulence factors, such as 2-heptyl-4-quinolone N-oxide (HQNO) (Fig. 1), pyocyanin, elastase, and rhamnolipids (9–13, 15, 20). While these molecules are known to interfere with *S. aureus* physiology, the mechanisms of action have not been fully elucidated.

**Fig 1:**
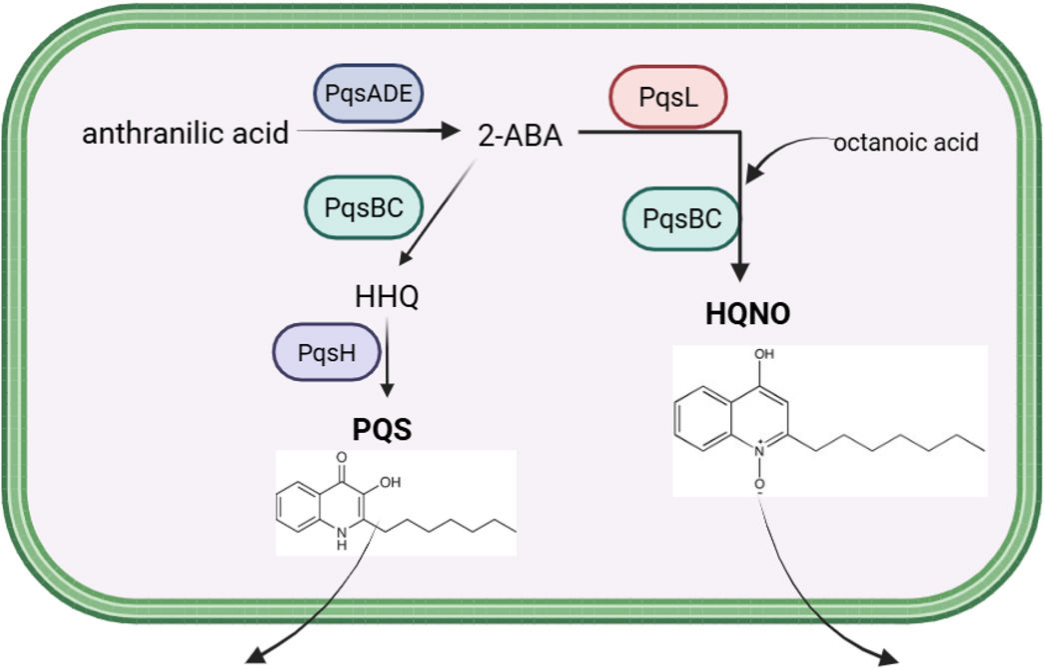
PQS and HQNO synthesis in *P. aeruginosa*. The PQS system of *P. aeruginosa* produces the secondary metabolites PQS and HQNO from anthranilic acid. The names of the enzymes responsible for the transformations are displayed. Abbreviations for the PQS intermediates are as follows: 2-aminobenzoylacetate (2-ABA), 2-heptyl-4-quinolone (HHQ), and 2-heptyl-4-quinolone N-oxide (HQNO). Created in BioRender. https://BioRender.com/j4lknb7.

The staphylococcal respiratory response regulator (SrrAB) is a two-component regulatory system (TCRS) that controls the transcription of genes utilized for respiratory and fermentative metabolisms (21–23). SrrB is a membrane-spanning histidine kinase/phosphatase (HK), and SrrA is a cytosolic response regulator (RR). The HK responds to a stimulus by altering the phosphorylation status of the RR. The RR has DNA-binding activity, and the affinity for DNA is altered by phosphorylation. The exact environmental and metabolic stimuli of the SrrAB system are unknown, but it is believed to sense the redox status of the menaquinone pool in a manner similar to the mechanism used by the *Escherichia coli* ArcAB system (24). SrrAB has increased activity during conditions where reduced menaquinone accumulates, and transcription of SrrAB-regulated genes is diminished in a menaquinone auxotroph (21). Upon decreased respiration, SrrAB increases transcription of the genes coding for the Cyd and Qox terminal oxidases, as well as genes coding for the anaerobic ribonucleotide reductase (Nrd), pyruvate formate lyase (Pfl), and lactate dehydrogenases (Ldh, Ddh) (25, 26).

We tested the hypothesis that the *S. aureus* SrrAB TCRS responds to respiratory inhibition caused by *P. aeruginosa* secreted secondary metabolites. Data presented demonstrate that SrrAB senses HQNO-dependent respiratory inhibition, and the presence of SrrAB increases survival when challenged with *P. aeruginosa. S. aureus* mutant strains with lesions in SrrAB-regulated genes that code for enzymes used for fermentation had decreased fitness and survival upon HQNO challenge and during co-culture with *P. aeruginosa*. Furthermore, we demonstrate that HQNO treatment of *S. aureus* increases the ratio of NAD^+^ to NADH, resulting in altered activity of the Rex transcriptional regulator. These findings provide a framework for further investigations into the physiological changes that *S. aureus* undergoes when challenged with *P. aeruginosa*.

## RESULTS

### *P. aeruginosa*-secreted secondary metabolites stimulate SrrAB and alter *srrAB* promoter activity independent of SrrAB

**Fig 2:**
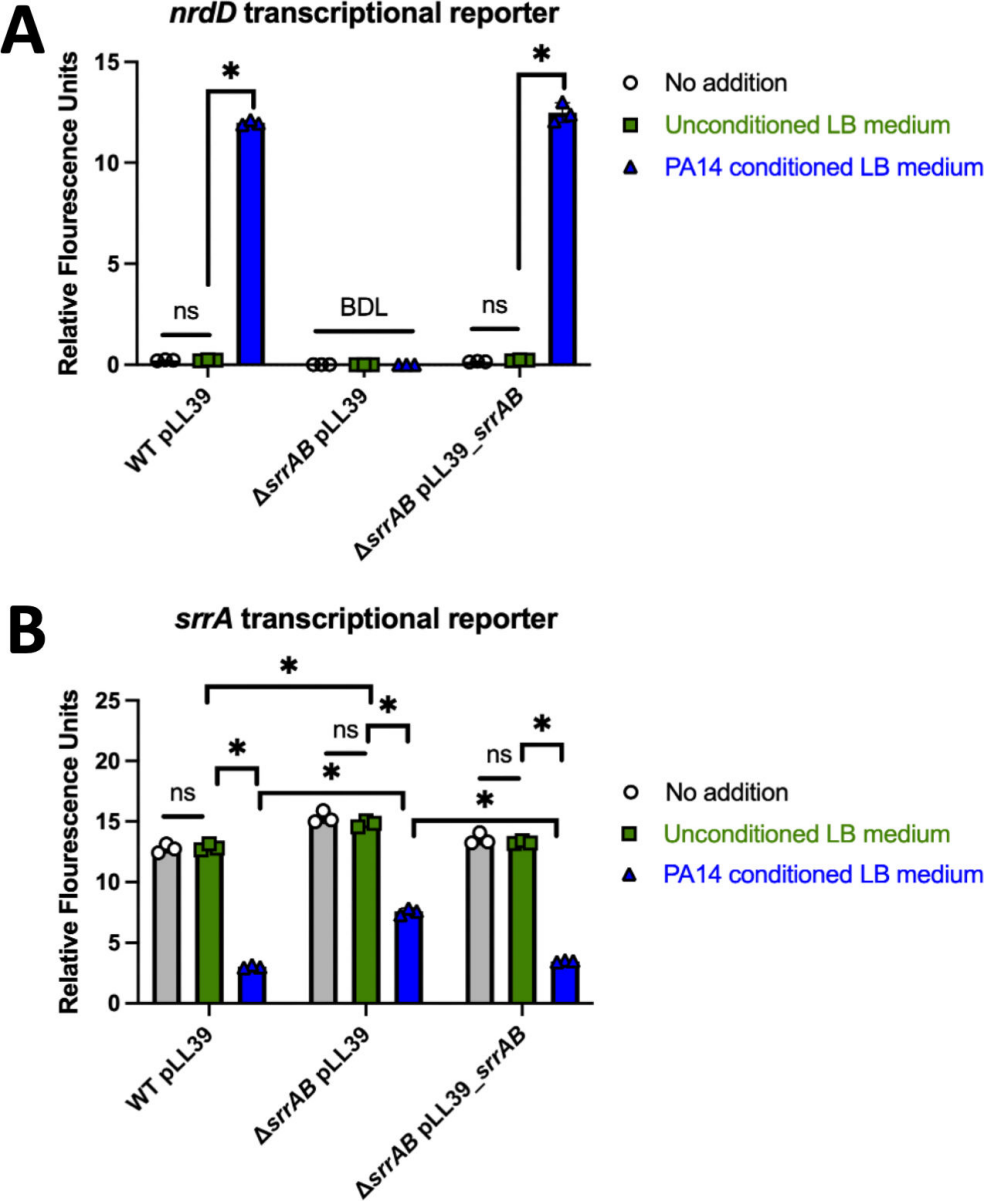
Effect of wild-type *P. aeruginosa* cell-free culture conditioned medium on SrrAB activity and *srrA* transcription. (**A and B**) Overnight cultures were diluted into fresh TSB-Cm and challenged with conditioned media for 8 h before *gfp* expression was quantified. The expression of *gfp* was monitored in the *geh::pLL39* (JMB1886), Δ*srrAB geh::pLL39* (JMB2276), and Δ*srrAB geh::pLL39_srrAB* (JMB 4746) strains containing the pOS_*nrdD_gfp* (**A**) or pOS_*srrA_gfp* (**B**) transcriptional reporter after growth in TSB-Cm with or without cell-free conditioned culture medium isolated from *P. aeruginosa* (PA14). Bars represent the averages of biological triplicates with standard deviations shown. The data for each individual strain in A were analyzed using a one-way analysis of variance (ANOVA), and *ad hoc* Tukey tests were conducted for pairwise comparisons. The data in B were analyzed using a two-way ANOVA, and *ad hoc* Tukey tests were conducted for pairwise comparisons. The * denotes a *P* value ≤0.05, and ns denotes no significant difference. BDL denotes below the detectable limit of the fluorimeter.

We tested the hypothesis that *P. aeruginosa* secreted secondary metabolites stimulate SrrAB. The gene coding for the iron–sulfur cluster requiring ribonucleotide reductase (*nrdD*) is transcriptionally regulated by SrrAB. It is one of the most highly upregulated genes when *S. aureus* transitions from respiratory to fermentative growth (26). *P. aeruginosa* (PA14) was cultured in LB medium overnight, and the conditioned cell-free culture medium was isolated and filter sterilized before being added to liquid cultures of *S. aureus* USA300_LAC parent strain or the isogenic Δ*srrAB* mutant. The strains contained either the pLL39 episome or the pLL39_*srrAB* episome integrated into the *geh* locus, and all strains contained a *nrdD* transcriptional reporter. There was a significant increase in *nrdD* promoter activity in the parent strain upon co-culture with the PA14 conditioned medium (Fig. 2A). The activity of the *nrdD* promoter was below the detectable limit in the Δ*srrAB* mutant, and this phenotype could be genetically complemented. Supplementing the medium with unconditioned LB did not alter *nrdD* promoter activity. These data suggest that SrrAB is required to transcribe *nrdD* and that one or more molecules are present in *P. aeruginosa* conditioned medium that stimulate SrrAB.

The *srrA* and *srrB* genes are encoded in an operon (*srrAB*), and the transcription of *srrAB* is positively autoregulated by SrrA during nitric oxide stress (27). We examined the effect of PA14 conditioned medium on the promoter activity of *srrA*. PA14 conditioned medium significantly decreased *srrA* promoter activity in the WT containing pLL39 (Fig. 2B). The activity of the *srrA* promoter was increased in the Δ*srrAB* mutant, but promoter activity was also decreased by co-culture with PA14 conditioned medium, suggesting that a transcription factor other than SrrAB was responsible for repressing *srrA* transcription. The increased *srrA* promoter activity in the Δ*srrAB* strain could be genetically complemented. These findings demonstrate that an altered abundance of one or more molecules in the *P. aeruginosa* conditioned medium impacts the output of the SrrAB TCRS and at least one additional transcriptional regulator.

**Fig 3:**
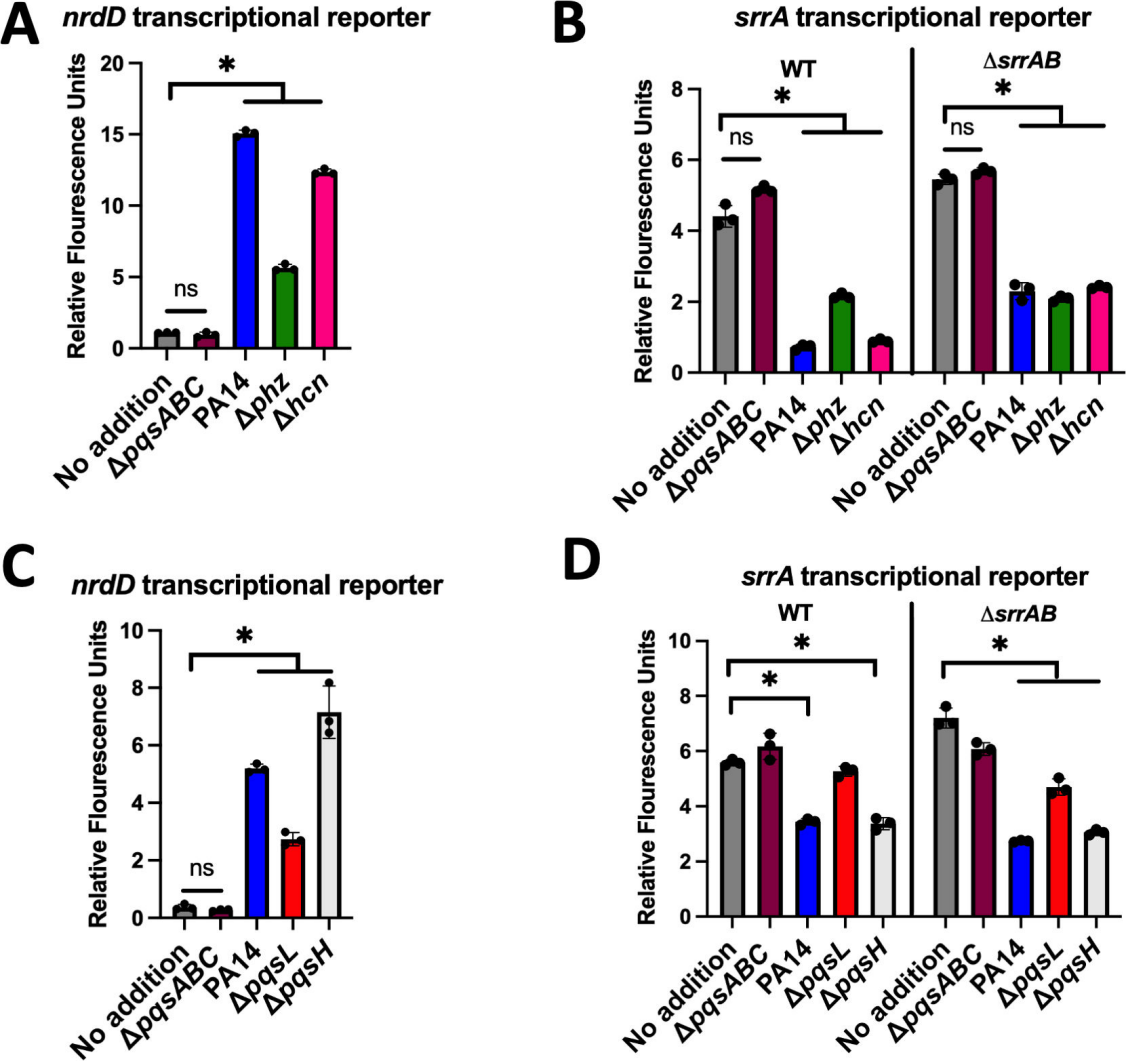
Effects of cell-free conditioned media isolated from different *P. aeruginosa* strains on SrrAB activity and *srrA* transcription. The expression of *gfp* was quantified in the wild-type (WT; JMB1100) and Δ*srrAB* (JMB1467) strains containing the pOS_*nrdD_gfp* (**A and C**) or pOS_*srrA_gfp* (**B and D**) transcriptional reporters after culture in TSB-Cm with or without cell-free conditioned culture media isolated from various isogenic *P. aeruginosa* strains. Each individual data bar corresponds to the average of the biological replicates, and standard deviations are shown, but the data points sometimes obscure them. The data in each panel and for each individual *S. aureus* strain were analyzed using a one-way ANOVA, and *ad hoc* Tukey tests were conducted for pairwise comparisons. The * denotes a *P* value ≤0.05, and ns denotes no significant difference.

We sought to determine which *P. aeruginosa* secondary metabolites were altering SrrAB regulatory output and *srrA* promoter activity. We harvested conditioned culture media from isogenic *P. aeruginosa* mutants that are deficient in producing one or more secondary metabolites. We then individually added these conditioned media to *S. aureus* and examined the activities of the *nrdD* and *srrA* promoters. The conditioned media from PA14, or mutants defective in either phenazine (Δ*phz*) or hydrogen cyanide (Δ*hcn*) production, increased *nrdD* promoter activity, suggesting that the production of these metabolites is not responsible for the altered SrrAB output (Fig. 3A). The conditioned culture medium harvested from the Δ*pqsABC* mutant failed to alter *nrdD* promoter activity (Fig. 3A). Conditioned medium harvested from the Δ*phz* mutant also had a diminished ability to stimulate *nrdD* promoter activity when compared to PA14, suggesting that one or more phenazines can directly or indirectly stimulate SrrAB.

Conditioned culture media from the PA14 or Δ*hcn* strains significantly decreased *srrA* promoter activity, whereas the conditioned medium from the Δ*pqsABC* mutant did not (Fig. 3B). Again, medium harvested from the Δ*phz* mutant had an intermediate phenotype compared to media harvested from the PA14 and Δ*pqsABC* strains. The Δ*srrAB* mutant also responded to the conditioned media isolated from the PA14, Δ*phz,* or Δ*hcn* strains, reinforcing the hypothesis that the PQS system produces one or more molecules that alter *srrAB* transcription independent of SrrAB. Because the conditioned medium from the Δ*pqsABC* mutant did not significantly alter *nrdD* or *srrA* promoter activity, we focused our attention on identifying and describing the metabolite derived from anthranilic acid (Fig. 1) that stimulated both SrrAB and a second transcriptional regulator. Future studies will examine the role of phenazines.

The *P. aeruginosa pqsL* and *pqsH* gene products are responsible for the production of HQNO and PQS, respectively (Fig. 1). We harvested conditioned culture media from isogenic strains incapable of generating one or more anthranilic acid-derived metabolites. Conditioned media from PA14, Δ*pqsL*, or Δ*pqsH* strains significantly increased *nrdD* promoter activity, whereas the conditioned medium from the Δ*pqsABC* mutant did not (Fig. 3C). The conditioned medium from the Δ*pqsL* had a decreased ability to stimulate *nrdD* transcription compared to PA14 conditioned medium. The conditioned medium from the Δ*pqsH* mutant caused a similar response to that seen with the PA14 medium.

The conditioned culture medium from the Δ*pqsL* mutant did not significantly decrease *srrA* promoter activity in the WT but did in the Δ*srrAB* mutant (Fig. 3D). Conditioned medium from the Δ*pqsH* strain decreased *srrA* promoter activity to a level like that seen with the PA14 conditioned medium. These results led to a model wherein *P. aeruginosa* produced HQNO alters SrrAB output, and modulated the activity of an additional regulatory protein that represses *srrA* transcription.

### HQNO stimulates SrrAB and leads to altered *srrA* transcriptional activity

**Fig 4:**
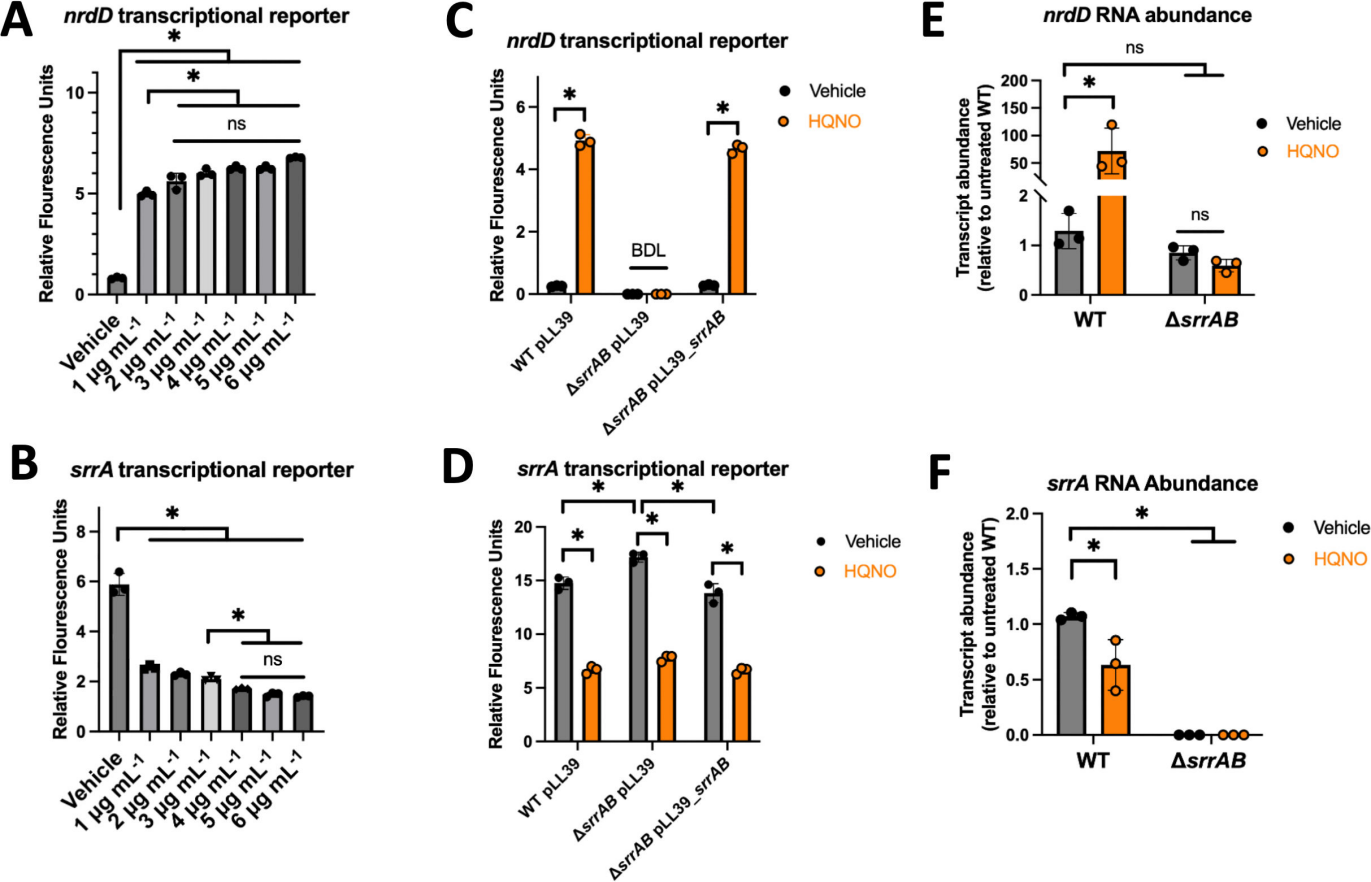
HQNO alters SrrAB activity and *srrA* transcription. (**A and B**) The wild-type (WT; JMB1100) strain containing the pOS_*nrdD_gfp* (**A**) or pOS_*srrA_gfp* (B) transcriptional reporters was co-cultured with different concentrations of HQNO or the vehicle control (DMSO) before *gfp* expression was quantified using fluorimetry. (**C and D**) The *geh*::pLL39 (JMB1886), Δ*srrAB geh*::pLL39 (JMB2276), and Δ*srrAB geh*::pLL39_*srrAB* (JMB4746) strains containing the pOS_*nrdD_gfp* (C) or pOS_*srrA_gfp* (**D**) transcriptional reporter were cultured in TSB-Cm with 5 µg mL^−1^ HQNO or vehicle before *gfp* expression was quantified using fluorimetry. (**E and F**) The abundances of mRNA transcripts corresponding to *nrdD* and *srrA* were quantified in the WT and Δ*srrAB* (JMB1467) strains using real-time quantitative PCR (qPCR) after culture in Tryptic soy broth (TSB) with 5 µg mL^−1^ HQNO or vehicle. Each individual data bar corresponds to the average of the biological replicates, and standard deviations are shown, but the data points sometimes obscure them. The data in A and B were analyzed using a one-way ANOVA, and *ad hoc* Tukey tests were conducted for pairwise comparisons. The data for each individual strain in C were analyzed using a two-tailed Student’s *t*-test. The data in D, E, and F were analyzed using a two-way ANOVA, and *ad hoc* Tukey tests were conducted for pairwise comparisons. The * denotes a *P* value ≤0.05, and ns denotes no significant difference. BDL denotes below the detectable limit of the fluorimeter.

We tested the hypothesis that HQNO stimulates SrrAB and alters the activity of the *srrA* promoter. We titrated pure HQNO into liquid cultures of the WT containing the *nrdD* transcriptional reporter and observed a significant dose-dependent increase in promoter activity (Fig. 4A). The increase in promoter activity plateaued at approximately 5 µg mL^−1^ HQNO. We next titrated HQNO into cultures of the WT containing the *srrA* transcriptional reporter. This resulted in a dose-dependent decrease in *srrA* promoter activity, with the maximal effect again occurring at approximately 5 µg mL^−1^ (Fig. 4B).

We sought to determine if the observed effects of HQNO on *srrA* and *nrdD* promoter activities require SrrAB. The activity of the *nrdD* promoter again increased in the parent strain (WT with pLL39) but did not increase in the Δ*srrAB* mutant strain with pLL39 upon treatment with HQNO, demonstrating a dependency on SrrAB (Fig. 4C). Returning *srrAB* to the Δ*srrAB* mutant resulted in a strain that phenocopied the parent strain. Consistent with previous results, the effect of HQNO on *srrA* promoter activity was independent of SrrAB (Fig. 4D).

To validate the data generated using the transcriptional reporters, we performed quantitative real-time PCR (qPCR) to measure the abundance of the *nrdD* and *srrA* transcripts. The *nrdD* transcript was significantly increased when challenged with HQNO compared to the cultured cells challenged with vehicle, while the *srrA* transcript showed a significant decrease (Fig. 4E and F). The data presented thus far are consistent with a model wherein *P. aeruginosa*-secreted HQNO alters SrrAB activity and *srrA* transcriptional activity via an alternative transcriptional regulator.

### Proficient respiration is required for HQNO to alter *srrA* transcription

**Fig 5:**
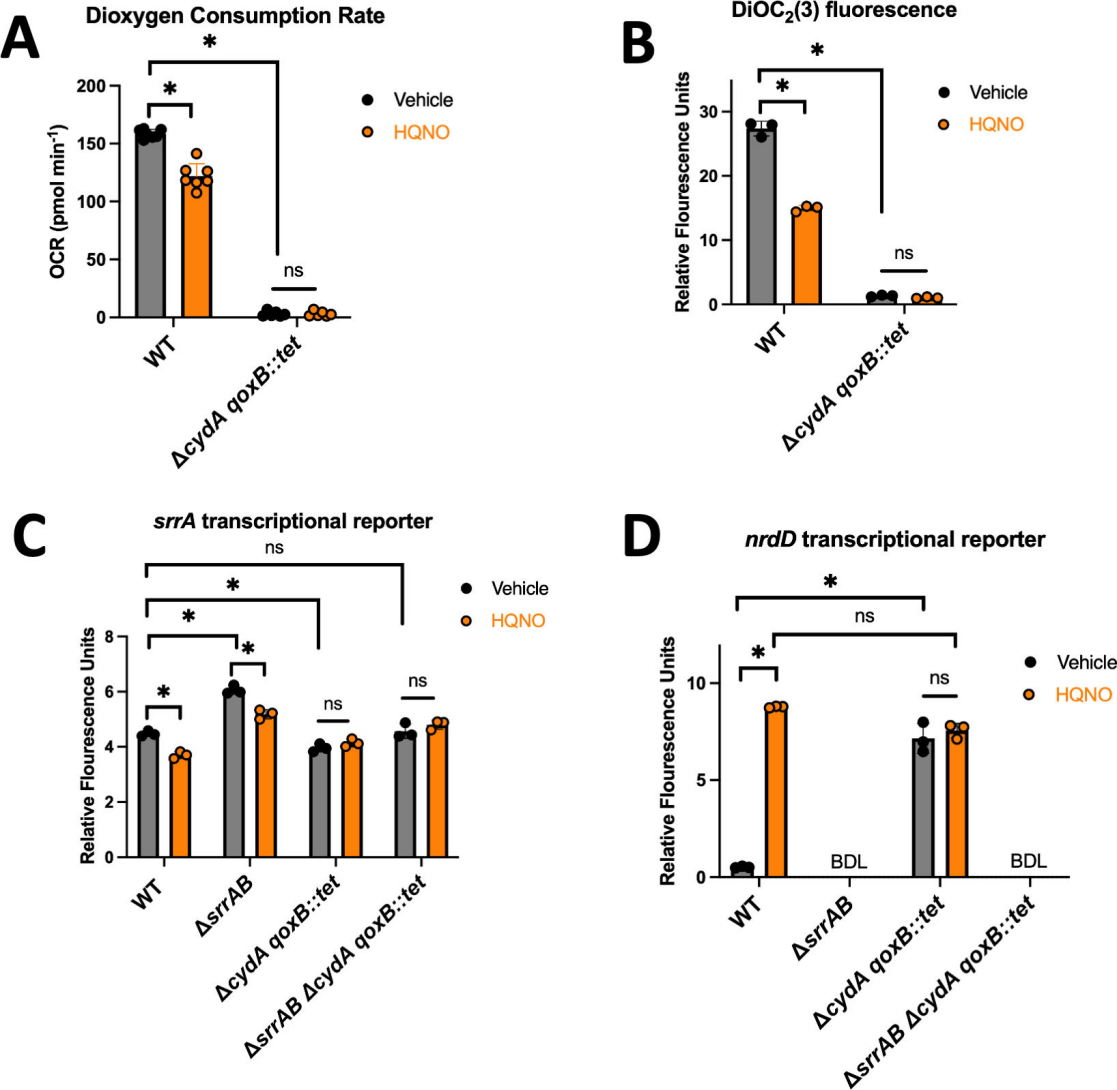
HQNO and dioxygen respiration. (**A**) Oxygen consumption rates were quantified in the wild-type (WT; JMB1100) and Δ*cydA qoxB::tet* (JMB 8988) strains after culture in TSB media with 5 µg mL^−1^ HQNO or vehicle control. (**B**) Membrane potential was assessed using 30 µM of the fluorescent dye DiOC_2_(3) in the WT and Δ*cydA qoxB::tet* strains after culture in TSB media with 5 µg mL^−1^ HQNO or vehicle control. (**C and D**) The WT, Δ*srrAB* (JMB1467), Δ*cydA qoxB::tet*, and Δ*srrAB* Δ*cydA qoxB::tet* (JMB 8989) strains containing pOS_*nrdD_gfp* or pOS_*srrA_gfp* after culture in TSB media with 5 µg mL^−1^ HQNO or vehicle control before *gfp* expression was quantified using fluorimetry. Each individual data bar corresponds to the average of the biological replicates, and standard deviations are shown, but the data points sometimes obscure them. The data in each panel were analyzed using a two-way ANOVA, and *ad hoc* Tukey tests were conducted for pairwise comparisons. The denotes a *P* value ≤0.05, and ns denotes no significant difference. BDL denotes below the detectable limit of the fluorimeter. The * denotes a *P* value ≤0.05, and ns denotes no significant difference. BDL denotes below the detectable limit of the fluorimeter.

It has been hypothesized that HQNO inhibits the cytochrome oxidases of *S. aureus,* resulting in the inhibition of respiration (9, 28). We tested the hypothesis that HQNO inhibited *S. aureus* respiration under the growth conditions used for our study. *S. aureus* utilizes two terminal oxidases called Cyd and Qox (29). We monitored oxygen consumption in the WT and a strain lacking functional terminal oxidases, after culture in the presence and absence of HQNO. The addition of HQNO significantly decreased the rate of dioxygen consumption in the WT (Fig. 5A). In contrast, no dioxygen consumption was noted for the Δ*cydA qoxB::tet* strain, and the presence of HQNO did not alter this phenotype.

We tested the hypothesis that challenging *S. aureus* with HQNO would decrease the membrane potential, which is a byproduct of respiration. The fluorescent dye DiOC_2_(3) can be used to compare the membrane potential of bacterial strains (30, 31). DiOC_2_(3) is positively charged and accumulates in a charge-dependent manner in the cells and shifts from green to red as the membrane potential increases (32). We treated aerobically cultured WT and Δ*cydA qoxB::tet* strains with HQNO and quantified DiOC_2_(3)-derived red fluorescence. Red fluorescence was lower in the Δ*cydA qoxB::tet* mutant than in the WT (Fig. 5B). The addition of HQNO decreased red fluorescence in the WT but did not alter red fluorescence in the Δ*cydA qoxB::tet* strain.

 We next tested the hypothesis that alterations in the activities of the *nrd* and *srrA* promoters upon HQNO addition require the cells to be respiring. We compared *srrA* promoter activity in the WT and Δ*cydA qoxB::tet* strains and their isogenic Δ*srrAB* mutants after aerobic culture with HQNO. We again noted that promoter activity decreased in the WT and Δ*srrAB* strains cultured with HQNO (Fig. 5C). The promoter activity of *srrA* was similar in the *cydA qoxB::tet* and *cydA qoxB::tet* Δ*srrAB* strains, consistent with a transcriptional regulator other than SrrA altering *srrA* promoter activity upon respiration inhibition. The activity of the *srrA* promoter was significantly decreased in the Δ*cydA qoxB::tet* strain compared to the WT but remained unchanged in the presence of HQNO. The activity of the *nrdD* promoter was increased in the Δ*cydA qoxB::tet* strain, and promoter activity was not affected by HQNO (Fig. 5D). These data are consistent with *srrA* and *nrdD* transcription being controlled by respiratory status, confirming that HQNO inhibits *S. aureus* dioxygen respiration and demonstrating that cells must be respiring for HQNO to alter SrrAB output and *srrA* transcription.

### Growth with HQNO alters metabolite pools, resulting in Rex repression of *srrAB* transcription

The data presented are consistent with a model wherein SrrAB and a second transcriptional regulator are acting on the *srrA* promoter when HQNO is present. We predicted that this second regulatory protein altered *srrA* promoter activity because of an altered metabolite pool upon HQNO-dependent respiration inhibition. We conducted an untargeted metabolomic analysis to identify metabolites that have altered abundances upon co-culture with HQNO. Several metabolites were significantly increased or decreased upon co-culture with HQNO (Table 1; Table S1). There were significant increases in NAD^+^, lactate, pyruvate, and acetyl-CoA, consistent with *S. aureus* increasing fermentation and decreasing TCA cycle activity to balance redox upon HQNO inhibition of dioxygen respiration. The concentrations of the amino acids alanine, glycine, threonine, and proline were significantly increased. Alanine can be synthesized from pyruvate via alanine dehydrogenases (29). Glycine is synthesized from threonine and/or serine, both of which were also increased in response to HQNO.

**TABLE 1:** Metabolites that are significantly altered in *S. aureus* upon growth with HQNO^*a*^

Metabolite	Log2 fold-change from non-treated
Citrulline	−5.6
NADPH	−3.9
Arginine	−2.2
Aspartate	−2.2
N-acetylaspartate	−1.8
NADP**^+^**	−1.4
Glutamate	−1.4
Coenzyme A	−1.3
Pyruvate	0.7
NAD**^+^**	0.8
Acetyl-CoA	1.0
Alanine	1.6
Ornithine	2.1
Glycine	2.4
Proline	2.6
Lactate	2.9
Threonine	3.9

^*a*^ All shown metabolites are statistically significant (two-tailed unpaired Student’s *t*-test *P* value < 0.05). For a full list of metabolites, refer to Table S1.

The metabolites that had the largest decrease upon co-culture with HQNO are intermediates in arginine synthesis or involved in nitrogen metabolism, including citrulline, aspartate, and glutamate (Table 1; Table S1). The one exception was ornithine, which was significantly increased with HQNO and could explain the increase in proline, as this amino acid is synthesized from ornithine (33). An additional explanation for these findings is the altered regulation of the *arcADRBC* operon, which codes for the arginine deiminase system. Arc functions in energy conservation during non-respiratory growth by importing arginine using an arginine-ornithine antiporter and then converting arginine to ornithine in the cytosol, generating ATP (34, 35).

**Fig 6:**
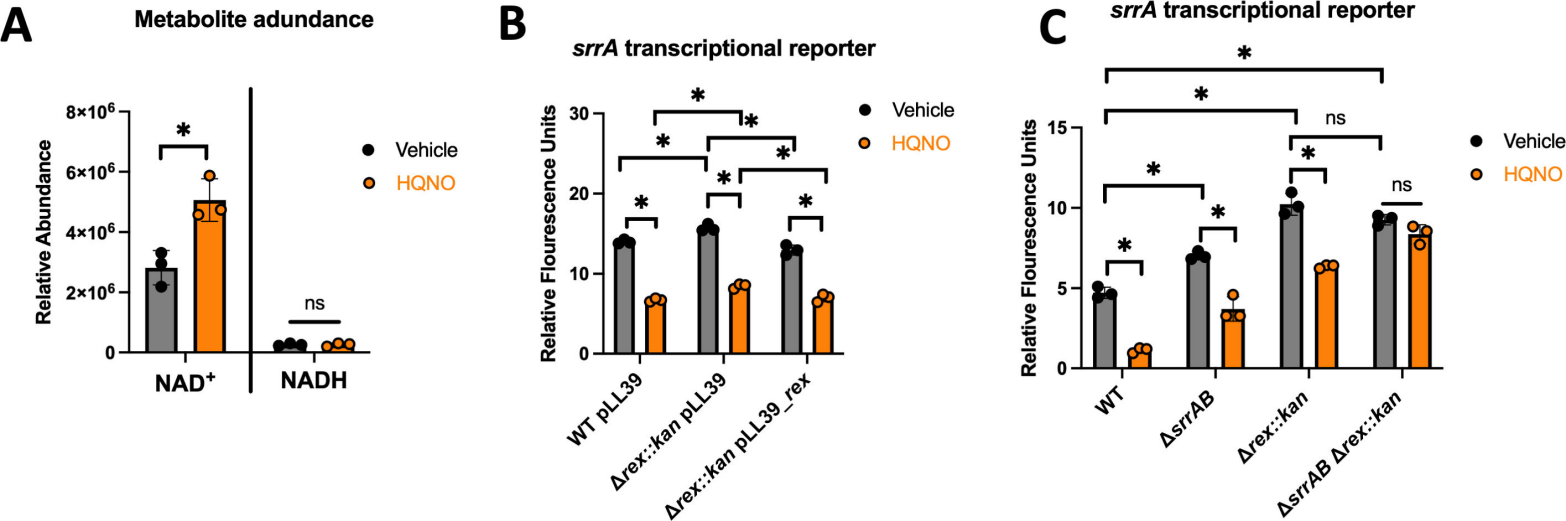
Rex represses *srrA* transcription in response to HQNO challenge. (**A**) The relative abundances of NAD^+^ and NADH in the wild-type (WT; JMB1100) strain after co-culture with 5 µg mL^−1^ HQNO or vehicle control. (**B**) The *geh::pLL39* (JMB1886), Δ*rex::kan pLL39* (JMB15637), and Δ*rex::kan pLL39_rex* (JMB15639) strains containing the pOS_*srrA_gfp* transcriptional reporters were co-cultured with HQNO (5 µg mL^−1^) or vehicle control (DMSO) before *gfp* expression was quantified using fluorimetry. (**C**) The expression of *gfp* was monitored in the WT, Δ*srrAB* (JMB1467), Δ*rex::kan* (JMB 13604), and Δ*srrAB* Δ*rex::kan* (JMB 13626) strains containing pOS_*srrA_gfp* after culture with 5 µg mL^−1^ HQNO or vehicle control. Each individual data bar corresponds to the average of the biological triplicates, and standard deviations are shown, but the data points sometimes obscure them. Two-tailed Student’s *t*-tests were used to compare the abundances of each metabolite in A. The data in B and C were analyzed using a two-way ANOVA, and *ad hoc* Tukey tests were conducted for pairwise comparisons. The * denotes a *P* value ≤0.05, and ns denotes no significant difference. BDL denotes below the detectable limit of the fluorimeter.

NAD^+^, but not NADH, increased upon HQNO treatment, resulting in an increase in the NAD^+^ to NADH ratio (Fig. 6A; Table 2). Similarly, there was an overall increase in oxidized nicotinamide adenine dinucleotide pools compared to their reduced counterparts (Table 2). Accumulation of NAD^+^ increases the affinity of the *S. aureus* transcriptional regulator Rex for DNA *in vitro* (27, 36). Rex controls the transcription of the *arc* operon, and there is a Rex binding site in the promoter of *srrA* that ends −85 base pairs upstream from the translational start site. Rex directly associates with the *srrA* operator *in vitro* (27, 36).

**TABLE 2:** Ratios for nicotinamides when HQNO is present^*a*^

Metabolites	No addition	HQNO
NAD^+^/NADH	10.9	19.4
NAD^+^ + NADP^+^/NADH + NADPH	6.2	19.2

^*a*^ For a full list of metabolites, refer to Table S1.

We tested the hypothesis that Rex was responding to growth in the presence of HQNO to repress *srrA* transcription. We cultured the parent (WT pLL39), Δ*rex*, and a complemented Δ*rex* strain (*rex* expressed from the *geh* locus containing the pLL39 episome) in the presence or absence of HQNO. As previously reported, the Δ*rex* mutant had increased *srrA* promoter activity when compared to the parent strain, and the phenotype could be genetically complemented, confirming that Rex represses *srrA* transcription (Fig. 6B) (27, 36). The addition of HQNO decreased *srrA* promoter activity similarly across all three strains. These data confirm that Rex represses the promoter activity of *srrA* but also suggest that a second transcriptional regulator is acting on the promoter.

We tested the hypothesis that both SrrA and Rex repress *srrA* transcription upon growth with HQNO. We quantified the activity of the *srrA* promoter in the WT, Δ*rex*, Δ*srrAB*, and Δ*rex* Δ*srrAB* strains after growth in the presence or absence of HQNO. The activity of the *srrA* promoter was increased in the Δ*rex,* Δ*srrAB*, and Δ*rex* Δ*srrAB* strains when compared to the WT (Fig. 6C). The phenotype of the Δ*rex* mutation was dominant, and the Δ*rex* Δ*srrAB* phenocopied the Δ*rex* strain. The addition of HQNO decreased *srrA* promoter activity in the WT, Δ*rex,* and Δ*srrAB* strains, but not in the Δ*rex* Δ*srrAB* strain. These findings demonstrate that Rex and SrrA respond to growth in the presence of HQNO and independently repress *srrA* transcription.

### SrrAB contributes to fitness and survival when *S. aureus* is exposed to HQNO or PA14

**Fig 7:**
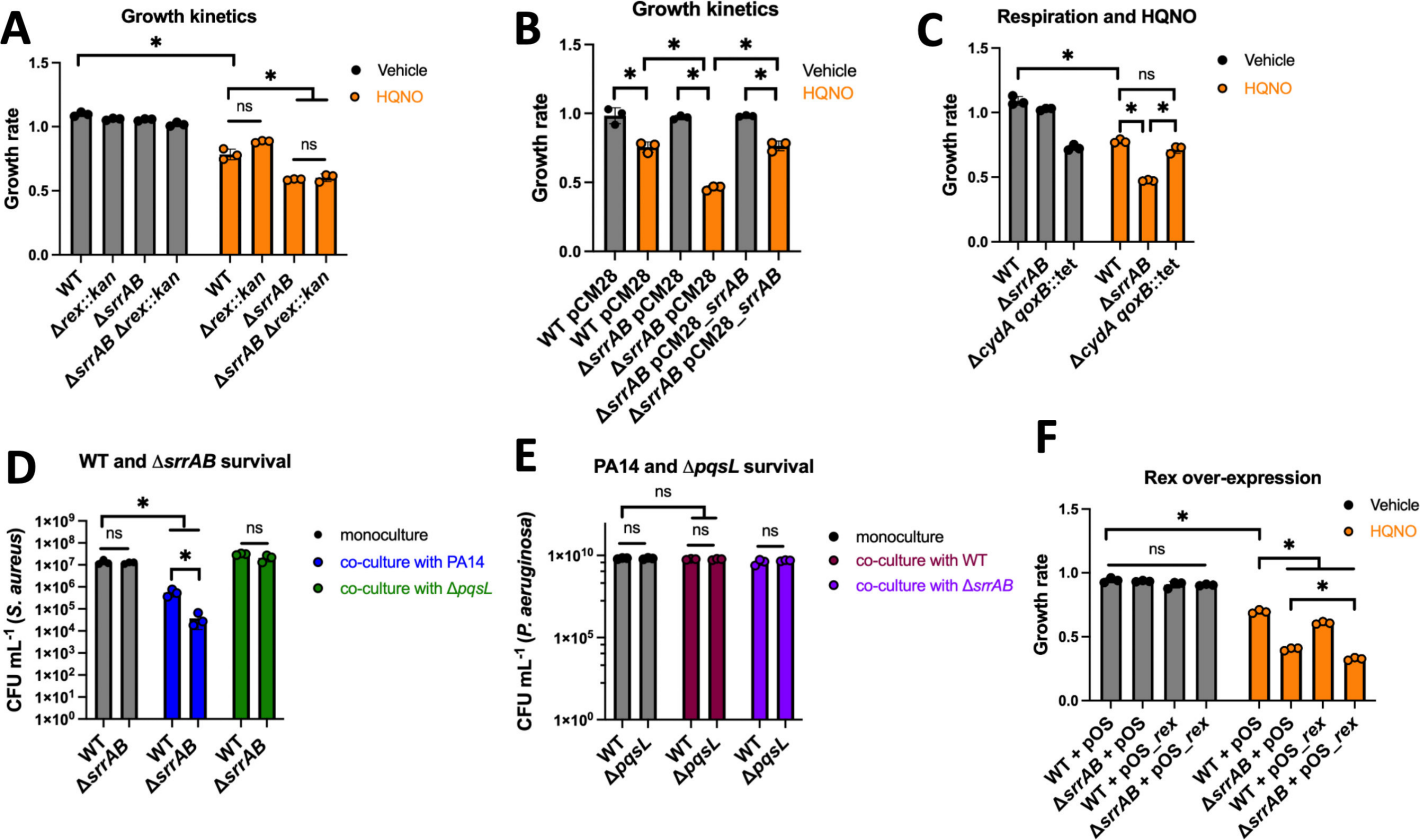
SrrAB is required for fitness and survival when challenged with HQNO and *P. aeruginosa*. (**A**) The growth rates for the wild-type (WT; JMB 1100), Δ*srrAB* (JMB1467), Δ*rex::kan* (JMB 13604), and Δ*srrAB* Δ*rex::kan* (JMB 13626) strains were quantified after growth in TSB media with 5 µg mL^−1^ of HQNO or vehicle control. (**B**) The growth rates of the WT or Δ*srrAB* strains containing pCM28 or pCM28_*srrAB* were quantified after growth in TSB media with 5 µg mL^−1^ of HQNO or vehicle control. (**C**) The growth rates for the WT, Δ*srrAB*, and Δ*cydA qoxB::tet* (JMB8988) strains were quantified after growth in TSB with 5 µg mL^−1^ of HQNO or vehicle control. (**D**) *P. aeruginosa* PA14 (JMB 10389) or isogenic Δ*pqsL* (JMB13827) mutant was co-cultured with the *S. aureus* WT or Δ*srrAB* mutant before *S. aureus* survival was determined by counting colony-forming units (CFU) after plating on TSB with 7.5% NaCl. (**E**) *P. aeruginosa* PA14 or isogenic Δ*pqsL* mutant was co-cultured with the *S. aureus* WT or Δ*srrAB* mutant before *P. aeruginosa* survival was determined by counting CFU after plating on TSB. (**F**) The growth rates of the WT and Δ*srrAB* containing either pOS1 or pOS1_*rex* were quantified after culture in TSB-Cm media with 5 µg mL^−1^ of HQNO or vehicle control. Each individual data bar corresponds to the average of biological triplicates, and standard deviations are shown, but the data points sometimes obscure them. The data in each panel were analyzed using a two-way ANOVA, and *ad hoc* Tukey tests were conducted for pairwise comparisons. The * denotes a *P* value ≤0.05, and ns denotes no significant difference.

We monitored growth kinetics to test the hypothesis that SrrAB and Rex promote fitness when challenged with HQNO. The WT, Δ*srrAB*, Δ*rex*, and Δ*srrAB* Δ*rex* mutants all had lower growth rates in Tryptic soy broth (TSB) containing HQNO, but the growth rate of the Δ*srrAB* mutant was lower than that of the treated WT, whereas the Δ*rex* mutant showed no such difference (Fig. 7A). The growth rate of the Δ*rex* Δ*srrAB* double mutant phenocopied that of the Δ*srrAB* strain. Expressing *srrAB* in multicopy using its native promoter genetically complemented the HQNO-challenged growth rate phenotype of the Δ*srrAB* mutant (Fig. 7B). These findings demonstrate that SrrAB is needed to maintain fitness when exposed to HQNO.

SrrAB is a positive transcriptional activator for genes utilized for respiration and fermentation, when respiration is impaired (25, 26). We further investigated the role of SrrAB in improving fitness and survival when challenged with HQNO. We cultured the WT, Δ*srrAB*, and Δ*cydA qoxB::tet* strains aerobically in the presence and absence of HQNO. The Δ*cydA qoxB::tet* strain had a lower growth rate than the WT strain, a difference that was unaffected by HQNO, consistent with HQNO inhibiting respiration (Fig. 7C). The growth rates of the WT and Δ*cydA qoxB::tet* strains were similar when challenged with HQNO, whereas the growth rate was lower for the Δ*srrAB* mutant. These data are consistent with a model wherein the role of SrrAB in increasing fitness when challenged with HQNO is more than just increasing the transcription of genes coding respiratory components.

*P. aeruginosa* can outcompete *S. aureus* when they are co-cultured. The finding that SrrAB was required for fitness of HQNO-challenged *S. aureus* led to the hypothesis that SrrAB is also essential for survival when co-cultured with *P. aeruginosa*. We quantified the survival of the WT and Δ*srrAB* strains with and without co-culture with strains of *P. aeruginosa* that can and cannot produce HQNO. Both *S. aureus* strains had decreased survival when co-cultured with *P. aeruginosa*, but the Δ*srrAB* mutant had a lower survival than the WT (Fig. 7D). The killing of the WT and Δ*srrAB* mutant was abrogated upon co-culture with the *P. aeruginosa* Δ*pqsL* mutant, demonstrating that HQNO was a primary determinant that allowed *P. aeruginosa* to prevent the proliferation of *S. aureus* in co-culture under the conditions tested. We also quantified *P. aeruginosa* survival and noted that co-culture with *S. aureus* did not impact the survival of either *P. aeruginosa* strain (Fig. 7E). These data demonstrate that SrrAB increases the ability of *S. aureus* to compete with an HQNO-producing strain of PA14.

Overexpression of Rex negatively impacts *S. aureus* growth under respiration-inhibiting conditions because it represses the transcription of genes utilized for fermentation, including those encoding lactate dehydrogenases (27). We created *S. aureus* strains with a plasmid containing *rex* under the transcriptional control of the constitutive *lgt* promoter (pOS_*rex*). The WT and Δ*srrAB* strains containing pOS_*rex* had a lower growth rate than those containing the empty vector (pOS) upon HQNO challenge (Fig. 7F). These data indicate that increasing the expression of *rex* reduces the ability of *S. aureus* to overcome the HQNO challenge.

### Effective fermentation promotes fitness and survival when *S. aureus* is challenged with HQNO or PA14

**Fig 8:**
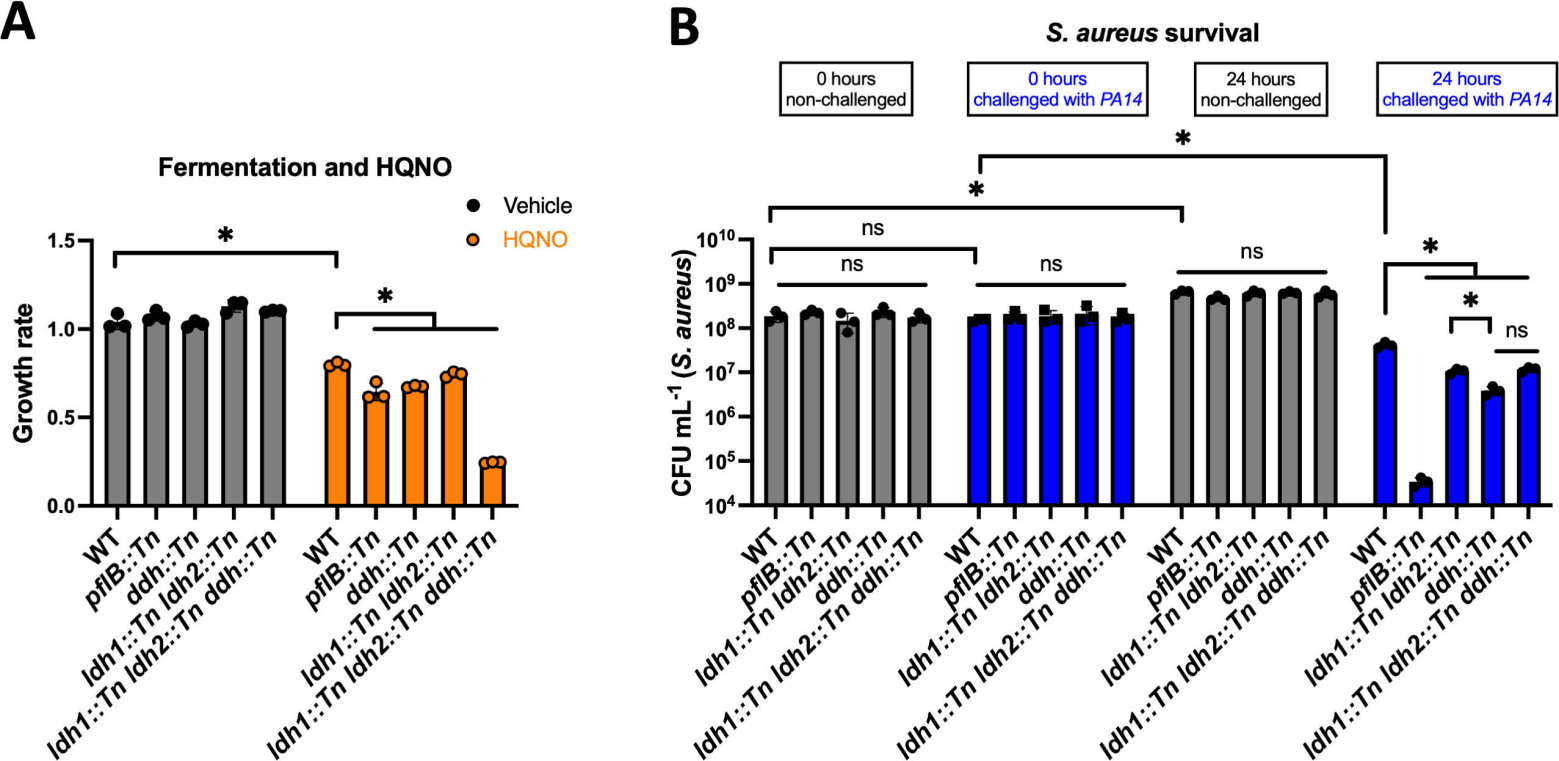
Effective fermentation is important for *S. aureus* fitness and survival upon HQNO or *P. aeruginosa*. (**A**) The growth rates of the wild-type (WT; JMB 1100), *pflB::Tn* (JMB 10737), *ddh::Tn* (JMB 14930), *ldh1::Tn ldh2::Tn* (JMB 14175), and *ldh1::Tn ldh2::Tn ddh::Tn* (JMB 14176) strains were quantified after growth in TSB media with 5 µg mL^−1^ of HQNO or vehicle control. (**B**) The same *S. aureus* strains used for A were individually cultured in TSB or in co-culture with *P. aeruginosa* PA14 (JMB 10389). The *S. aureus* CFU were quantified upon inoculation and after 24 h of plating on TSB with 7.5% NaCl. Each individual data bar corresponds to the average of the biological replicates, and standard deviations are shown, but the data points sometimes obscure them. The data in both panels were analyzed using a two-way ANOVA, and *ad hoc* Tukey tests were conducted for pairwise comparisons. The * denotes a *P* value ≤0.05, and ns denotes no significant difference.

We next tested the hypothesis that effective fermentation is required for fitness upon HQNO challenge. SrrAB regulates the genes coding lactate dehydrogenases and Pfl (25–27). During fermentative growth, Pfl is utilized to produce acetyl-CoA (37). We determined that the growth rate of a *pflB::Tn* mutant was significantly reduced compared to the WT when cultured with HQNO (Fig. 8A).

Lactate, generated from pyruvate, is a primary fermentation byproduct in *S. aureus*, and it accumulates upon treatment with HQNO (Table 1). The *S. aureus* genome codes for two cytosolic L-lactate dehydrogenases (*ldh1*, *ldh2*), a cytosolic D-lactate dehydrogenase (*ddh*), and a quinone-linked lactate dehydrogenase (*lqo*) (38). We examined the growth kinetics of the WT, *ldh1::Tn ldh2::Tn*, *ddh::Tn*, and *ldh1::Tn ldh2::Tn ddh::Tn* strains with and without HQNO. All strains had a significant decrease in growth rate when exposed to HQNO. The growth rates of the *ldh1::Tn ldh2::Tn* double mutant and *ddh::Tn* strains were lower than that of the WT, with the *ddh::Tn* mutant showing a stronger phenotype (Fig. 8A). The HQNO-dependent growth phenotypes associated with the *ldh1::Tn ldh2::Tn* mutations and the *ddh::Tn* mutation had a synergistic effect upon HQNO challenge. These data highlight the importance of using lactate to balance redox upon HQNO-dependent inhibition of respiration.

We hypothesized that the survival of *S. aureus* strains defective in fermentation would be decreased compared to the WT when co-cultured with *P. aeruginosa*. We quantified the survival of the WT, *pflB::Tn*, *ldh1::Tn ldh2::Tn*, *ddh::Tn*, and *ldh1::Tn ldh2::Tn ddh::Tn* strains before and after 24 h mono-culture or co-culture with PA14. The fermentation-deficient strains had decreased survival compared to the WT when they were co-cultured with PA14 (Fig. 8B). When grown in monoculture, there was an increase in colony-forming units (CFU) for all strains from the time of inoculation to 24 h post-inoculation, demonstrating that the strains are actively growing in the conditions utilized (Fig. 8B). There was no significant difference in CFU between the mono-cultured *S. aureus* strains, demonstrating that the decreased *S. aureus* CFU noted upon co-culture with PA14 was not the result of a strain-specific decreased growth rate. All mutant strains lacking one or more genes that are regulated by SrrAB and utilized during fermentation had decreased survival during co-culture when compared to the WT. These findings demonstrate that effective fermentation is required for fitness and survival when *S. aureus* is challenged with *P. aeruginosa*.

## DISCUSSION

*P. aeruginosa* secretes secondary metabolites that negatively impact the fitness of *S. aureus* (12, 14). The decrease in fitness leads to an increased ability of *P. aeruginosa* to outcompete *S. aureus* in a co-culture and, by an unknown mechanism, to stimulate cell death. This initial study aimed to determine if *S. aureus* senses the presence of *P. aeruginosa* secondary metabolites and, if so, to determine whether the regulatory response improves *S. aureus* fitness in co-culture. In *S. aureus*, SrrAB increases the transcription of genes utilized for fermentation and respiration when the respiration rate decreases (22, 23, 25). The results herein demonstrate that the SrrAB TCRS senses and responds to the presence of *P. aeruginosa*-conditioned medium. We used *P. aeruginosa* mutant strains and pure compound to determine that the PQS quorum-sensing system-derived molecule HQNO was responsible for stimulating SrrAB. Previous studies have demonstrated that during co-culture with *P. aeruginosa*, HQNO is one of the secondary metabolites involved in driving *S. aureus* toward a fermentative phenotype (17). Importantly, HQNO has been detected in cystic fibrosis patients infected with *P. aeruginosa,* and our results demonstrate that SrrAB promotes *S. aureus* survival when these two bacteria are co-cultured in the laboratory (16, 39). Conditioned medium isolated from a mutant incapable of producing phenazines also resulted in a partial induction of the *nrdD* promoter, suggesting that SrrAB is also capable of responding to the presence of these metabolites. Previous studies have demonstrated that the *P. aeruginosa*-produced phenazine pyocyanin can inhibit the *S. aureus* cytochrome *bd* quinol oxidase (CydAB) and, at high concentrations, select for non-respiring small colony variants (10, 40). It is plausible that the SrrAB regulatory system also senses phenazine-dependent respiratory inhibition, but additional studies are required to test this hypothesis.

**Fig 9:**
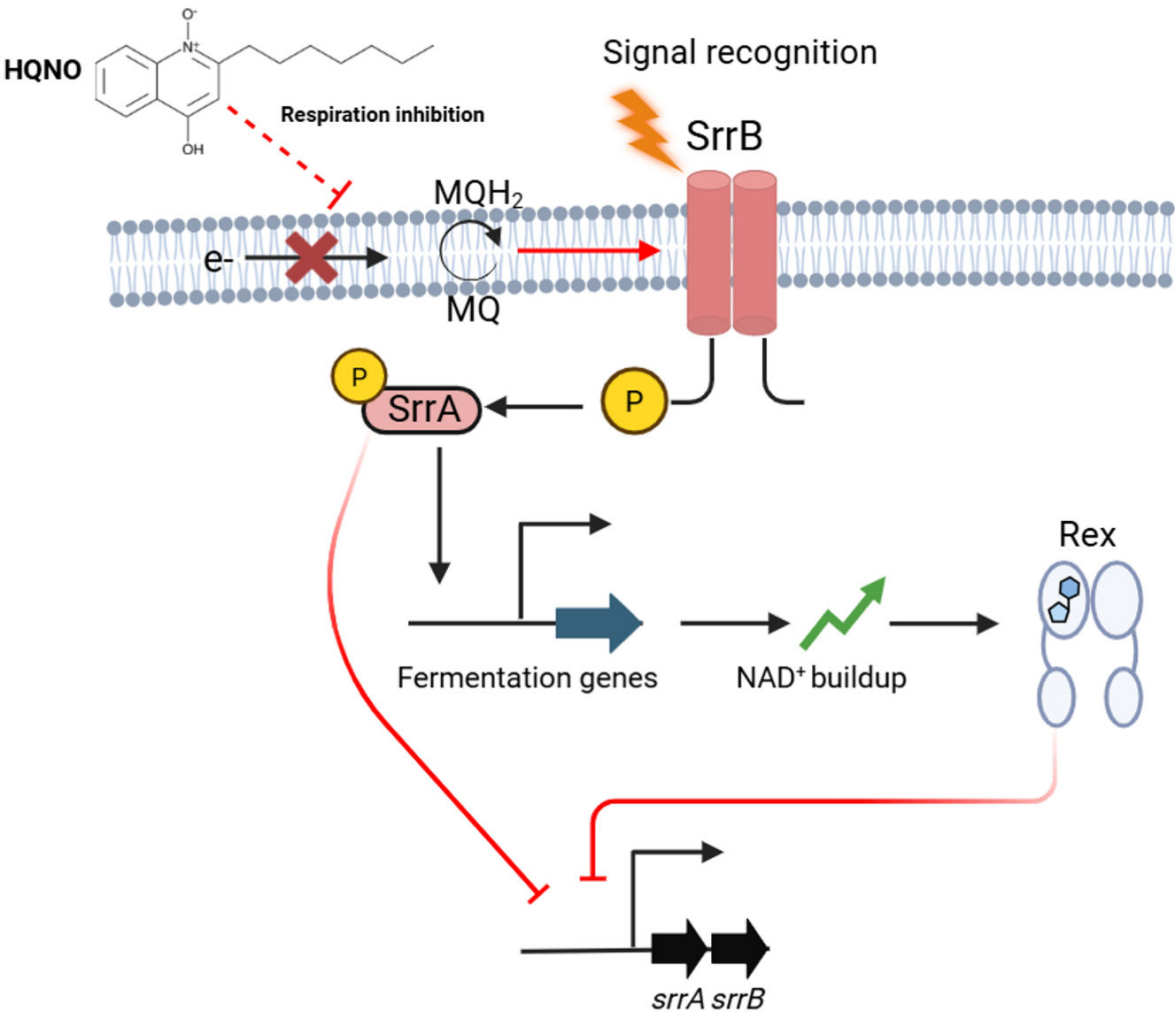
Proposed model for SrrAB regulation by Rex. *P. aeruginosa*-produced HQNO inhibits dioxygen respiration in *S. aureus,* stimulating the histidine kinase SrrB, which alters the titers of phosphorylated SrrA. SrrA then represses *srrAB* transcription and increases the transcription of genes utilized for fermentative and anaerobic growth. This exacerbates a fermentation phenotype and increases the ratio of NAD^+^ to NADH. The increased abundance of NAD^+^ binds to Rex, increasing its affinity for the operator of *srrAB* and repressing its transcription. Created in BioRender. https://BioRender.com/x36momp.

Previous work in our lab determined that either chemical or genetic inhibition of respiration increased SrrAB activity (21). Herein, we determined that treatment of WT *S. aureus* with HQNO resulted in decreased dioxygen consumption, a decreased proton motive force, and a decreased growth rate, whereas a mutant lacking both terminal oxidases showed no such effects. The activities of the *srrA* and *nrdD* promoters are regulated by SrrAB, and the activities of these promoters, upon treatment with HQNO, mimicked the phenotypes of the mutant strain lacking the terminal oxidases. These data support the working model in Fig. 9, where *P. aeruginosa*-generated HQNO inhibits *S. aureus* respiration, which is sensed by SrrAB, which responds by altering the transcription of genes utilized for fermentation. Consistent with this model, previous studies discovered that the co-culture of *P. aeruginosa* and *S. aureus* increased the transcription of SrrAB-regulated genes (30, 36).

HQNO is structurally similar to menaquinone, which is the lipid-soluble electron carrier that *S. aureus* utilizes to shuttle electrons between respiratory components (41). It has been hypothesized that one mechanism for HQNO-dependent inhibition of *S. aureus* respiration is competing with menaquinone for binding to NADH:oxidoreductase (NDH-2). HQNO decreased the rate of *S. aureus* NDH-2 reoxidation after NADH-dependent FAD reduction with a K_i_ app value of 6.8 ± 0.4 μM (42). The *Caldalkalibacillus thermarum* NDH-2 was inhibited with an IC_50_ of 7.3 μM in the presence of 50 μM menadione, which is a water-soluble menaquinone analog (43). Challenging *S. aureus* with HQNO has been linked to increased biofilm formation, a trait of anaerobically (fermentatively) cultured *S. aureus* (21, 44–46). We chose to work with a strain lacking the terminal oxidases because there are numerous electron inputs to the respiratory chain, including succinate dehydrogenase, malate quinone oxidoreductase, and quinone-linked lactate dehydrogenase, but only two enzymes that can transfer these electrons to dioxygen. Further biochemical analyses will be required to determine the precise respiratory component(s) that are inhibited by HQNO.

We discovered that the promoter activity of *srrA* was repressed by SrrA under the growth conditions utilized. A study by Dmitriev et al. also determined that SrrA autoregulated *srrAB* promoter activity, but they found that SrrA positively regulated *srrAB* transcription (27). They also determined that treatment of *S. aureus* with nitric oxide, a respiration inhibitor, stimulated SrrAB, resulting in increased *srrAB* transcription, whereas we found that inhibiting respiration decreases *srrAB* transcription. It is currently unknown why differences in this autoregulatory circuit are occurring, but we have described strain-dependent differences in SrrAB-dependent gene regulation in the past (22, 47). Dmitriev et al. used strain COL for their studies, whereas we used strain LAC. Our studies also used a different medium (TSB vs brain heart infusion) and a different culturing method that likely resulted in differences in the basal rate of respiration.

To our surprise, the activity of the *srrA* promoter was decreased in a Δ*srrAB* mutant in response to HQNO treatment. Three findings led to the hypothesis that Rex was responsible for this phenotype. First, metabolomic analyses found that the concentrations of metabolites associated with nitrogen metabolism were the most altered upon HQNO treatment. The concentrations of citrulline and arginine were decreased, while ornithine increased. These data suggested altered regulation of the *arcABCD* operon, which functions in energy conservation during non-respiratory growth. The *arcABCD* operon is directly regulated by Rex (36). Second, HQNO treatment increased the NAD^+^/NADH ratio, and increasing this ratio enhances the DNA-binding activity of Rex (36). Third, work by others determined that Rex associates with the *srrA* operator, and *srrA* transcription increases in a *rex* mutant (27, 36). Consistent with our hypothesis, epistasis experiments presented herein demonstrated that both SrrA and Rex repress *srrA* transcription, and HQNO treatment did not decrease *srrA* transcription in the Δ*srrAB* Δ*rex* double-mutant strain.

When the NAD^+^/NADH ratio rises, the affinity of Rex for DNA increases, resulting in repressed transcription of genes involved in fermentation, including those coding lactate dehydrogenases, alcohol dehydrogenases, and Pfl (27, 36). Rex also represses *srrAB* transcription, which is likely because SrrAB stimulation increases the transcription of genes involved in fermentation (21, 23). It initially seemed counterintuitive that inhibition of respiration would increase the ratio of NAD^+^ to NADH, resulting in increased Rex activity; however, the experiments presented examined promoter and metabolomic changes that occurred after prolonged exposure to HQNO, providing the necessary time for *S. aureus* to adjust to respiration inhibition. Consistent with this, growth with HQNO increased lactate accumulation, which coincides with past findings demonstrating that co-culturing of *S. aureus* with *P. aeruginosa* increased the production of lactate and alcohol dehydrogenases (17). Rex and SrrA regulate the expression of *ldh* and *adh* genes (36, 48).

Upon respiratory inhibition, SrrAB stimulation increased the transcription of genes involved in fermentation (21, 25, 26). We determined that SrrAB, and not Rex, was important for *S. aureus* survival when co-cultured with *P. aeruginosa*. The slow-growth phenotype associated with the Δ*srrAB* mutation during co-culture with HQNO was exacerbated in a respiration-deficient strain, likely because SrrAB is also an activator of transcription of genes utilized for fermentation. Consistent with this, mutations in fermentation genes reduced the growth rate and survival of *S. aureus* during co-cultures with *P. aeruginosa. S. aureus* mutants lacking lactate dehydrogenases had lower growth rates and survival when compared to the WT. This coincides with a previous report in which *P. aeruginosa* consumes the lactate secreted by *S. aureus* during fermentation (17). A *S. aureus* strain lacking functional Pfl (*pflB*) also had lower survival when compared to the other fermentation mutants. Previous studies done by Filkins et al. determined that *pflB* was one of the most upregulated genes in *S. aureus* during co-culture with *P. aeruginosa* and HQNO challenge (17).

Inhibition of respiration increased Rex-dependent repression of *srrA*. The Δ*rex* mutant had a growth rate like WT when challenged with HQNO. This is likely because Rex is a repressor, and the transcription of genes that promote fermentation was alleviated in the Δ*rex* strain. In support of this hypothesis, increased expression of *rex* decreased the growth rate when HQNO was present, presumably because of increased Rex repression of genes utilized for fermentative redox balance. This finding correlates with a previous study demonstrating that Rex overexpression decreased *S. aureus* growth when respiration was inhibited by nitric oxide (27). The finding that the ratio of NAD^+^/NADH increased suggested increased fermentation upon HQNO treatment. Increased production of lactate dehydrogenases, which oxidize NADH, is a primary mechanism to balance redox during fermentation. The decreased growth rates in the lactate dehydrogenase mutant strains upon HQNO addition demonstrated the importance of redox balance upon HQNO-dependent respiratory inhibition (Fig. 8A).

The results of this study demonstrate how *P. aeruginosa*-secreted HQNO inhibits respiration in *S. aureus,* resulting in a regulatory feedback loop. Respiratory inhibition is sensed by the SrrAB TCRS, which responds by increasing the transcription of genes involved in fermentation and redox balance. This favors the oxidation of NADH to NAD^+^, activating Rex, which represses *srrAB* transcription. The inability of *S. aureus* to sense respiration inhibition and respond using SrrAB, or to utilize Pfl or lactate dehydrogenases, decreases cell fitness and survival when co-cultured with HQNO or *P. aeruginosa*, respectively. Future studies using infection models and the integration of host physiology are necessary to determine how these findings shed light on *S. aureus* and *P. aeruginosa* competition in patients suffering from cystic fibrosis.

## MATERIALS AND METHODS

### Bacterial strains and experimental conditions

* *TSB, purchased from VWR, was used for liquid growth analyses. The liquid media was supplemented with 1.5% agar (VWR) to make solid media. Unless stated differently, aerobic cultures were grown in 2 mL of TSB in 10 mL culture tubes, shaken at 200 rpm at 37°C. Tubes were slanted at a 30 degree angle to promote aeration. Cells were treated with 0–5 µg mL^−1^ HQNO (Selleck Chemicals) prepared as a 5 mg mL^−1^ stock in DMSO or DMSO as the vehicle control. Plasmids or chromosomal insertions were selected using antibiotics at final concentrations of 30 µg mL^−1^ chloramphenicol or 10 µg mL^−1^ for erythromycin (Erm), kanamycin (Kan), and tetracycline (Tet). Plasmids were maintained by supplementing the media with 10 µg mL^−1^ Cm (TSB-Cm). DNA primers were purchased through Integrated DNA Technologies (Coralville, IA).

### Plasmids and construction of bacterial strains

All plasmids and bacterial strains utilized for this study are listed in Table 3. All transductions were performed using bacteriophage 80α (49). All *S. aureus* strains used were isogenic to erythromycin-susceptible USA300_LAC (wild type; WT) (50). Bacterial strains and plasmids were PCR-verified and subsequently sequenced by Azenta (South Plainfield, NJ). Amplicons were generated using USA300_LAC DNA as a template and DNA primer pairs (Table 4). For transcriptional reporter construction, amplicons were digested with *KpnI-*HF and *HindIII-*HF (New England Biolabs) for 1 h at 37°C, followed by heat inactivation at 80°C for 20 min and ligated into similarly digested pOS_*saeP1_gfp* (51) using Quick Ligase (New England Biolabs). The ligation product was transformed into *E. coli* DH5-α and selected for growth on lysogeny broth (LB) agar plates containing 1 µg mL^−1^ ampicillin. To create the pLL39_*rex* complementing episome, pLL39 was digested with BamHI and SalI, and an amplicon was generated using the following primer pair: Rex5BamHI and Rex3SalI. The amplicon was digested and ligated into pLL39. Colonies were verified for the presence of the insert by PCR, and plasmids were extracted. Plasmids were transformed into *S. aureus* RN4220 (52), and cells were selected for Tet (pLL39) resistance. Episome integration was verified as outlined elsewhere (53). Plasmids and episomes were transduced into USA300_LAC background using bacteriophage α80 (49).

**TABLE 3:** Strains and plasmids used in this study

Name	Genotype	Source
*Pseudomonas aeruginosa* strains		
JMB 10389	PA14 (wild type)	(54) Lars Dietrich
JMB 10393	Δ*pqsABC*	(55) Lars Dietrich
JMB 10391	Δ*phz*	(56) Lars Dietrich
JMB 10394	Δ*hcn*	(57) Lars Dietrich
JMB 13827	Δ*pqsL*	(55) Lars Dietrich
JMB 13828	Δ*pqsH*	(55) Lars Dietrich
*Staphylococcus aureus* strains		
JMB 1100	USA300_LAC (wild type)	(50)
JMB 1467	Δ*srrAB*	(21)
JMB 8988	Δ*cydA qoxB::tet*	This study
JMB 8989	Δ*cydA qoxB::tet* Δ*srrAB*	This study
JMB 2047	Δ*srrAB::tet*	(21)
JMB 13604	Δ*rex::kan*	This study (27)
JMB 13626	Δ*srrAB* Δ*rex::kan*	This study
JMB 10737	*pflB::Tn*	This study (58)
JMB 14930	*ddh::Tn*(*kan*)	This study (58, 59)
JMB 14175	*ldh1::Tn*(*erm*) *ldh2::Tn*(*spec*)	This study (58, 59)
JMB 14176	*ldh1::Tn*(*erm*) *ldh2::Tn*(*spec*) *ddh::Tn*(*kan*)	This study (58, 59)
JMB 1886	*geh::pLL39*	(60)
JMB 2276	Δ*srrAB geh::pLL39*	(21)
JMB 4746	Δ*srrAB geh::pLL39_srrAB*	(21)
JMB 15637	Δ*rex::kan geh::pLL39*	This study
JMB 15639	Δ*rex::kan geh::pLL39_rex*	This study

Plasmid name	Plasmid function	Source
pOS	Cloning vector	(61)
pOS_*nrdD*_*gfp*	*nrdD* transcriptional reporter	This study
pOS*_srrA_gfp*	*srrAB* transcriptional reporter	This study
pOS_*rex*	Rex overexpression plasmid from *lgt* promoter	(27)
pCM28	Empty cloning vector	Horswill
pCM28_*srrAB*	*srrAB* complementing vector	(21)
pLL39	Genetic complementation	(53)
pLL39_*rex*	*rex* genetic complementation	This study

**TABLE 4:** Primers used for this study

Name	Sequence	Source
pOS_nrd_hindIII	gggaagcttGATGGGCATTGTGACGATTTAAGTATG	This study
pOS_nrd_kpnI	gcgggtaccATATATTGTGTCTACTCATTACG	This study
pOS_srr_hindIII	gggaagcttGTGATGCGTCATTTAGCAGAACATGG	This study
pOS_srr_kpnI	gcgggtaccACAGGTCATACCTCCCACACATGCTTT	This study
GFP verification	GTGTCCATTTACATCTCCGTCAAGTTC	This study
nrdDpSalI up	gggGTCGACGATGGGCATTGTGACGATTTAAGTATG	This study
nrdDpmulI down	gggACGCGTATATATTGTGTCTACTCATTACG	This study
ldh1 Fwd	GGGAAGCTTGCATTGTGTACAATTGTCTG	This study
ldh1 Rev	GCAAATCGCCTCTGCTAGACAATC	This study
ldh2 Fwd	GTACTATTTTTTGCTAATTTTCTAACAA	This study
ldh2 Rev	ATGCATACGGTACTTTCTCATCGCCTTT	This study
ddh Fwd	GGGAAGCTTGCTATACAAATTATACTTC	This study
ddh Rev	CGTGTTTCACATGTACCAGTGTTAATG	This study
srrA For	GCAGAACATGGGAAATAATTAAATAAAATATGTATTTATCACAAAG	This study
srrA Rev	aCAGGTCATACCTCCCACACATGCTTT	This study
Rexveri3	CCTGAGACATACTTAGTCCCAG	This study
Rexveri5	CAGTATAAAACGCCGTCTTGAAAC	This study
Rex5BamHI	GGGGGATCCCCATTTTACAGTATAAAACGCCGTCTTG	This study
Rex3SalI	CCCGTCGACGGCGATTAATTACGATTCATCATTATATC	This study

### Kinetic growth analyses

* *To assess the impact of *P. aeruginosa* on *S. aureus* fitness, we cultured *S. aureus* strains in triplicate overnight in TSB and then diluted them. To an optical density (A_600_) of 0.1 in 2 mL of TSB in 10 mL culture tubes. Two sets of triplicates per strain were prepared, and 5 µg mL^−1^ HQNO was added to one set. A total of 200 µL from each prepared set of triplicates was added to a 96-well plate. To avoid evaporation, the culture wells on the edge of the plate were not used and sterile water was added to the empty wells around the experimental wells so that all wells had 200 µL of liquid to minimize evaporation. The 96-well plate and lid were sealed with parafilm around the meeting joint to avoid evaporation. The strains were grown in a Biotek Epoch 2 Microplate Reader for approximately 16 h with constant shaking (shake speed 2) at 37°C, and OD_600_ readings were taken every 15 min. The culture optical densities (A_600_) were standardized to the media only blanks, and data were plotted as log10 culture optical density vs time. Two data points were selected that lay within the linear region (logarithmic plot), which were read 1 h apart (t1 and t2), and growth rates were determined using the following equation:


$$Growth\;Rate=\ln\left(\frac{Optical\;density\;t2}{Optical\;density\;t1}\right)$$

### Transcriptional reporter assays

Strains were grown overnight in TSB containing 10 µg mL^−1^ Cm and subsequently diluted to an optical density (A_600_) of 0.1 in 2 mL of TSB-Cm in 10 mL culture tubes. After 8 h of growth, 200 µL of each sample was added to black 96-well plates (Thermo-Scientific), and fluorescence emission was measured at a 488 nm excitation, 510 nm emission, using a Varioskan Lux plate reader (Thermo Scientific). For assays with *P. aeruginosa* conditioned medium, *P. aeruginosa* strains were grown overnight in 3 mL of LB in a 30 mL culture tube. Cultures were centrifuged at 13,000 × *g* for 1 min, followed by filter sterilization of supernatants using 0.22 µm syringe filters (VWR). After *S. aureus* dilution, 100 µL of the *P. aeruginosa* conditioned media was added to 2 mL *S*. *aureus* culture. For assays utilizing HQNO, a final concentration of 0–10 μg mL^−1^ (typically 5 µg mL^−1^) of HQNO was added to *S. aureus* cultures that had been diluted to 0.1 (A_600_) in 2 mL of TSB-Cm in 10 mL culture tubes. Sample fluorescence was standardized to the culture optical densities (A_600_) at harvest time.

### Assessment of membrane potential

Overnight cultures were diluted 1:100 in a 10 mL culture tube with 2.5 mL of TSB and grown for 6 h at 37°C with constant shaking. Two milliliters of cells were pelleted by centrifugation. Cells were washed twice with PBS before diluting cells to an optical density (A_600_) of 0.085, and 200 µL were added in triplicate to black 96-well plates. 3,3′-Diethyloxacarbocyanine iodide [DiOC_2_(3)] was added to a final concentration of 30 µM. The plate was incubated statically at 37°C in the dark for 30 min. Fluorescence was then measured (450 nm excitation, 650 nm emission).

### Oxygen consumption rate

To assess the oxygen consumption rate (OCR), samples were analyzed using a Seahorse XFe96 Extracellular Flux Analyzer (Agilent Technologies) as previously described (62). Briefly, overnight cultures were diluted 1:100 into 10 mL of TSB media in 125 mL flasks and cultured with or without 5 µg mL^−1^ HQNO at 37°C with shaking at 300 rpm for 4 h. Cells were then diluted to an optical density (A_600_) of 0.025, and 200 µL was added into each well of a 96-well cell culture microtiter plate. The Seahorse analyzer was calibrated by hydrating the XFe sensor cartridge with sterile water overnight in a non-CO_2_ incubator at 37°C. Pre-warmed XFe calibrant was added to the wells 2 h before measurement. The OCR and ECAR were measured through 15 cycles with 3 min of measurements and 3 min of mixing.

### Metabolomics and sample preparation

#### Sample preparation

Triplicates of *S. aureus* LAC were cultured overnight in TSB before diluting to 0.1 (A_600_) in 5 mL of TSB in 25 mL culture tubes. Cells were grown for an additional 8 h with 5 µg mL^−1^ HQNO or vehicle control, after which 1 mL of each sample was centrifuged at 13,000 × *g* for 1 min and washed once with PBS. Cell pellets were resuspended in 1 mL of an ice-cold 2:2:1 solution of methanol:acetonitrile:H_2_O. Harvested cells were lysed in a FastPrep homogenizer (MP Biomedicals) with 600 µL of 0.1 mm lysing matrix beads (MP Biomedicals) (two cycles, 40 s, 6.0 m s^−1^). Samples were incubated on ice for 5 min before centrifuging twice at 13,000 × *g* for 2 min at 4°C and retaining the supernatant. A total of 850 µL of the supernatant was filtered through a 13 mm 0.2 µM (Pall Acrodisc) syringe filter. The samples were frozen at −80°C until transferred to the metabolomics core of the Rutgers Cancer Institute of New Jersey for analysis.

#### UHPLC chromatography conditions

The HILIC separation was performed on a Vanquish Horizon ultra-high-performance liquid chromatography (UHPLC) system (Thermo Fisher Scientific, Waltham, MA) with XBridge BEH Amide column (150 mm × 2.1 mm, 2.5 µm particle size, Waters, Milford, MA) using a gradient of solvent A (95%:5% H_2_O:acetonitrile with 20 mM acetic acid, 40 mM ammonium hydroxide, pH 9.4), and solvent B (20%:80% H_2_O:acetonitrile with 20 mM acetic acid, 40 mM ammonium hydroxide, pH 9.4). The gradient was as follows: 0 min, 100% B; 3 min, 100% B; 3.2 min, 90% B; 6.2 min, 90% B; 6.5 min, 80% B; 10.5 min, 80% B; 10.7 min, 70% B; 13.5 min, 70% B; 13.7 min, 45% B; 16 min, 45% B; 16.5 min, 100% B and 22 min, 100% B (63). The flow rate was 300 µL min^−1^. Injection volume was 5 µL and column temperature was 25°C. The autosampler temperature was set to 4°C and the injection volume was 5 µL.

#### Full scan mass spectrometry

The full scan mass spectrometry analysis was performed on a Thermo Q Exactive PLUS with a HESI source which was set to a spray voltage of −2.7 kV under negative mode and 3.5 kV under positive mode. The sheath, auxiliary, and sweep gas flow rates were set to 40, 10, and 2 (arbitrary unit), respectively. The capillary temperature was set to 300°C and aux gas heater was 360°C. The S-lens RF level was 45. The m/z scan range was set to 72 to 1,000 m/z under both positive and negative ionization mode. The AGC target was set to 3e6, and the maximum integration time (IT) was 200 ms. The resolution was set to 70,000.

#### Data analysis and quality

The full scan data were processed with a targeted data pipeline using the MAVEN software package (64). The compound identification was assessed using accurate mass and retention time match to the metabolite standards from the in-house library.

Prior to running the samples, the liquid chromatography–mass spectrometry system was evaluated for performance readiness by running both a commercially available standard mixture and an in-house standard mixture to assess the mass accuracy, signal intensities, and retention time consistency. All known metabolites in the mixture were detected within 5 ppm mass accuracy. Method blank samples matching the composition of the extraction solvent were used in every sample batch to assess background signals, and it was ensured that there was no carryover from one run to the next. In addition, the sample queue was randomized with respect to sample treatment to eliminate the potential for batch effects. The data can be accessed via the MassIVE database, under accession number MSV000099005.

### *S. aureus* survival assays

The survival assays were prepared by growing overnight cultures of *S. aureus* in 2 mL of TSB in 10 mL culture tubes and *P. aeruginosa* strains in 3 mL of LB in a 30 mL culture tubes. These cultures were then diluted 1:100 in triplicate in their respective growth media and cultured for an additional 2.5 h. Culture optical densities (A_600_) were then standardized to an optical density (A_600_) of 0.4 in 1 mL of TSB. The *P. aeruginosa* strains were further diluted 1:1,000 (1 µL) into the culture tubes containing 1 mL of a *S. aureus* strain (A_600_ 0.4). The bacteria were then co-cultured at 37°C for 24 h with agitation. After 24 h, the co-cultures were centrifuged at maximum speed for 2 min, and the supernatant was discarded. The cell pellets were resuspended in PBS and repeated two additional times. The washed cells’ optical densities (A_600_) were measured and then standardized to an optical density of 0.1 in PBS. A serial dilution up to 10^−7^ was performed for each of the co-cultures, and 5 µL of each dilution was spotted on TSB plates containing 7.5% (wt/vol) NaCl to inhibit *P. aeruginosa* growth. The plates were incubated at 37°C for 24 h, and CFU were determined.

### Quantitative real-time PCR assay

Bacterial strains were grown overnight in TSB and diluted to an optical density (A_600_) of 0.1 into 5 mL of TSB with or without 5 µg mL^−1^ HQNO in 30 mL culture tubes in triplicate. Cells were grown for 8 h at 37°C with constant agitation, after which they were pelleted by centrifugation 13,000 × *g* for 1 min. Cell pellets were treated with RNAprotect (Qiagen), and washed in 0.5 mL PBS, pH 7.4, and resuspended in 100 µL of 50 mM Tris, pH 8, containing 6.7 µg lysostaphin and 6.7 µg DNAse. Cells were then incubated at 37°C for 30 min. A total of 200 µL of a lysis buffer solution (20 mM sodium acetate, 1 mM EDTA, 0.5% SDS, 13.4 µg lysostaphin) was added to the samples and incubated at 65°C for 5 min. RNA extraction and isolation were done as previously described (65). The cDNA library was constructed using the High-Capacity cDNA Reverse Transcription Kit (Biosystems). A StepOnePlus thermal cycler (Applied Biosystems) performed the quantitative real-time PCR. The transcripts were standardized to transcripts corresponding to the 16S RNA gene (SAUSA300_1841; *rrsC*). All data sets were analyzed using the comparative C_T_ method (66, 67).

## Data Availability

The dataset includes raw and processed LC-MS data, standard mixtures for system validation, method blanks, and sample files used for metabolite detection. All data are available via the MassIVE database, under accession no. MSV000099005.

## SUPPLEMENTAL MATERIAL

Table S1 jb.00101-25-s0001.xlsx Metabolite abundances.

ASM does not own the copyrights to Supplemental Material that may be linked to, or accessed through, an article. The authors have granted ASM a non-exclusive, world-wide license to publish the Supplemental Material files. Please contact the corresponding author directly for reuse.
